# Role of nanotechnology and gene delivery systems in TRAIL-based therapies

**DOI:** 10.3332/ecancer.2016.660

**Published:** 2016-08-01

**Authors:** George E Naoum, Fady Tawadros, Ammad Ahmad Farooqi, Muhammad Zahid Qureshi, Sobia Tabassum, Donald J Buchsbaum, Waleed Arafat

**Affiliations:** 1 Alexandria Comprehensive Cancer Centre, Egypt; 2 East Tennessee State University, 1276 Gilbreath Dr, Johnson City, TN 37604, USA; 3 Institute of Biomedical and Genetic Engineering (IBGE), Islamabad, Pakistan; 4 University of Alabama at Birmingham, 1720 2nd Ave S, Birmingham, AL 35233, USA; 5 University of Alexandria, El-Gaish Rd, Egypt, Alexandria, Egypt

**Keywords:** TRAIL, nanotechnology, cancer gene therapy, apoptosis

## Abstract

Since its identification as a member of the tumour necrosis factor (TNF) family, TRAIL (TNF-related apoptosis-inducing ligand) has emerged as a new avenue in apoptosis-inducing cancer therapies. Its ability to circumvent the chemoresistance of conventional therapeutics and to interact with cancer stem cells (CSCs) self-renewal pathways, amplified its potential as a cancer apoptotic agent. Many recombinant preparations of this death ligand and monoclonal antibodies targeting its death receptors have been tested in monotherapy and combinational clinical trials. Gene therapy is a new approach for cancer treatment which implies viral or non-viral functional transgene induction of apoptosis in cancer cells or repair of the underlying genetic abnormality on a molecular level. The role of this approach in overcoming the traditional barriers of radiation and chemotherapeutics systemic toxicity, risk of recurrence, and metastasis made it a promising platform for cancer treatment. The recent first Food Drug Administration (FDA) approved oncolytic herpes virus for melanoma treatment brings forth the potency of the cancer gene therapy approach in the future. Many gene delivery systems have been studied for intratumoural TRAIL gene delivery alone or in combination with chemotherapeutic agents to produce synergistic cancer cytotoxicity. However, there still remain many obstacles to be conquered for this different gene delivery systems. Nanomedicine on the other hand offers a new frontier for clinical trials and biomedical research. The FDA approved nanodrugs motivates horizon exploration for other nanoscale designed particles’ implications in gene delivery. In this review we aim to highlight the molecular role of TRAIL in apoptosis and interaction with cancer stem cells (CSCs) self-renewal pathways. Finally, we also aim to discuss the different roles of gene delivery systems, mesenchymal cells, and nanotechnology designs in TRAIL gene delivery.

## Introduction

The symphony of balance between cell growth and cell death,in order to preserve normal biological functional environment, is maintained through orchestrated processes. Among these processes is apoptosis which is programmed cell death [1]. Countless researchers have highlighted that any interference with this balance can lead to cancer development [2]. The process of apoptosis itself is still an extremely complicated process in which many molecules and cellular pathways are involved [3]. In this review we aim to focus on TRAIL, a leading molecule involved in the process of apoptosis and its promising effects as apoptosis inducer for cancer treatment. We also aim to highlight the role of different gene delivery systems as alternative approaches to conventional TRAIL-based therapies in cancer.

## TRAIL a holy grail to apoptosis

Since the discovery of TNF family members, a new milestone in apoptosis has emerged. During the 1990s, Wiley *et al* and Pitti *et al* identified a new member of the TNF named TRAIL (tumour necrosis factor-related apoptosis-inducing ligand) or Apo 2 ligand [4, 5]. The major biological role of this 281–amino acid type II *trans*-membrane protein is apoptosis induction, after interacting with its receptors [6–8]. Four different homologous human TRAIL receptors have been classified, these are TRAIL-R1/DR4, TRAIL-R2/ DR5 also known as killer, TRAIL-R3 or DcR1, and TRAIL-R4 or DcR2 [9–12]. The death receptors DR4 and DR5, both contain a preserved death domain (DD) motif and play an important role in signal apoptosis signalling [13, 14]. The other receptors TRAIL-R3/DcR1 and TRAIL-R4/DcR2 have homology to the DR4 and DR5 extracellular domains and are believed to act as decoys binding to TRAIL and inhibit its effect on apoptosis induction [15].

After binding to its receptor, TRAIL activates two main signalling pathways; the intrinsic and extrinsic pathways for cell death [16].

### The extrinsic pathway

**Figure 1.**
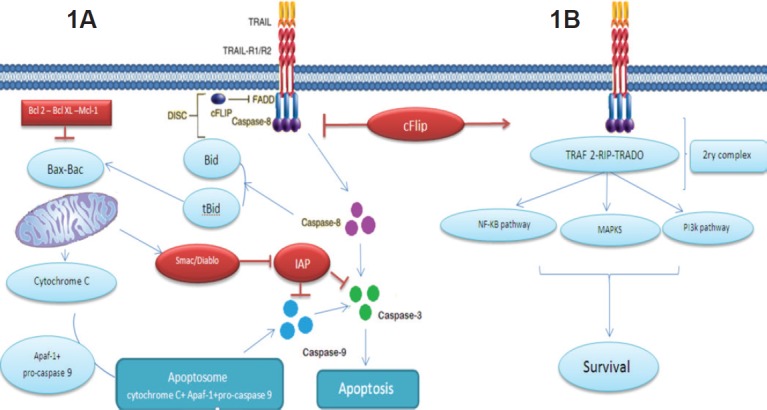
Showing the TRAIL signalling pathway. On the right Figure(1A), the apoptotic pathway induced by TRAIL and to the left Figure (1B), the resistance to TRAIL induced apoptosis. Binding of TRAIL and trimerisation of TRAIL death receptors leads to recruitment of FADD, an adaptor molecule that is capable of caspase-8 recruitment and activation. Apoptosis is either induced through direct caspase-8-mediated caspase-3 activation or through an amplification loop involving the mitochondria and the cleavage of the BH3-only protein Bid by caspase-8, cFLIP interferes with the generation of active caspase-8, attenuating the role of DISC.

The cell apoptotic extrinsic pathway is initiated when TRAIL binds three receptor molecules of either DR5 or DR4 forming homotrimers, each at the interface between two of its subunits [17–18], like most other TNF family members. This ligation and receptor trimerisation results in aggregation of the receptor’s intracellular DD, leading to the formation of a macromolecular complex known as death-inducing signalling complex (DISC); where an adaptor molecule Fas associated death domain protein (FADD) is recruited and binds to caspase-8 and -10 to activates them. The activated caspase-8 and -10 allow the conversion of pro-caspase-3 to its active form which in turn leads to cleavage of the death substrates. It is to be noted that this pathway can be inhibited by the cellular FLICE inhibitory protein (c-FLIP). This protein shares homologous sequence with caspase 8, and thus might compete with it for FADD binding in the DISC, as illustrated in Figure 1B. The presence of the c-FLIP protein in the DISC attenuates its function as it lacks protease activity. In addition to that, in the presence of c-FLIP, DISC forms a secondary complex with receptor-interacting protein (RIP), NFκB kinase, TNFR1-associated death domain (TRADD), and TNF receptor-associated factor 2 (TRAF2). This secondary complex activates non-apoptotic signals initiated through the mitogen-activated protein kinase (MAPK) pathways, nuclear factor κB, and phosphoinositide 3-kinase (PI3K)/Akt.

On the other hand, the two apoptosis signalling pathways,the intrinsic and the extrinsic, communicate with each other [19] as depicted in Figure 1A.

### The intrinsic pathway

Upon death stimulus, Bid (a member the proapoptotic Bcl-2 family) is cleaved by caspase-8. This cleavage leads to the formation of truncated form of Bid that interact with Bax and Bak in mitochondria to destabilise its outer membrane, so cytochrome *c* can be released. In the cytosol, an apoptosome is formed by the binding of cytochrome *c* to the adaptor APAF-1 and pro-caspase-9 as depicted in Figure 1A. In turn, caspase-9 after being activated by the apoptosome activates ‘executioner’ protease caspase -3, -6, and -7. This connection apparently plays a role in amplifying the response to death receptor activation and different types of cells might rely more on this amplification pathway than others [20]. This pathway was found to be inhibited by members of IAP (inhibitory of apoptosis) family including cellular IAP 1 and 2, X-linked IAP, and survivin. These molecules contribute to TRAIL resistance by inhibiting the activity of caspases 3, 7, and/or 9. Nevertheless, Smac (second mitochondria-derived activator of caspases)/DIABLO (direct inhibitor of apoptosis with low pi) which is released from mitochondria by Bax during apoptosis can antagonise their effect [21]. This role explains why Bax inactivation or mutation in mismatch-repair (MMR)-deficient tumours can be responsible for TRAIL resistance [7, 22]. Also, Bax introduction in Bax deficient cells restored TRAIL sensitivity [21]. Recent studies also indicate that combination of TRAIL with chemotherapy or radiation can overcome TRAIL resistance by overexpression of Bax [7, 23–24].

Interestingly these TRAIL apoptotic effects tend to be more abundant in cancer cells compared to normal cells [25–27].

## TRAIL in oncology field

### The promising cancer apoptotic inducer

Multiple cancer cells have developed mechanisms to evade a tightly regulated cell death programme, rendering a more aggressive pattern to the disease. These mechanisms include inactivating proapoptotic cell death components or manipulating the levels of antiapoptotic molecules [28]. Thus, it is unsurprising that using naturally present apoptosis inducer molecules in cancer treatment has become a reasonable approach. Among these molecules used in induction of apoptosis is the Fas ligand [29] and members of TNF family [30]. When compared to other TNF family members and Fas ligand TRAIL showed more safety as TNF and Fas induce cytotoxicity against tumour cells with a lethal inflammatory response caused by the first and severe hepatotoxicity caused by the second in murine models [31]. The balance between TRAIL efficiency associated with selectivity to cancer cells and potential safety made it a powerful tool in cancer treatment [32–33]. Also, the molecular pathway of TRAIL spotted it as a promising apoptosis induction agent ahead of other chemotherapeutic formulations. The supremacy of TRAIL over most DNA-damaging drugs also lies in its ability to induce apoptosis in different cell lines regardless the p53 status [34]. These chemotherapeutics depend solely on p53 for intrinsic apoptosis pathway induction and hence the molecular pathway of TRAIL provides a solution circumventing tumour treatment resistance acquired through mutations in p53. Added to that, another attractive feature is that combination of chemotherapeutics and TRAIL lead to synergism in apoptosis induction [35]. Interestingly, it is to be noted that ionising radiation (IR), a mainstay regimen in many cancer types treatment, can sensitise cancer cells to TRAIL action as well [36]. This synergism has been attributed to many molecular and cell signalling changes [3, 24, 37].

This synergism can be used in a TRAIL combinational approach to reach maximum apoptosis in cancer. Another consequential benefit from this synergism is the usage of lower doses of either chemotherapeutics or radiation and hence limiting their well-known systemic side effects. Another additive feature of TRAIL over these agents is its ability to affect cancer stem cells.

### TRAIL effects in eradicating cancer stem cells

Cancer stem cells (CSCs), have been proposed to be responsible for the self-renewal, growth of tumours, and their recurrence after treatment owing to the analogy that maintenance of adult normal tissues is mediated through tissue-specific SCs [38–39]. Therefore the new era in cancer therapies lies in targeting and killing CSCs. Like other tumour cells, CSCs possess death receptors (DR) that can be targeted by TRAIL [40]. It was found that DR are expressed at a high level in glioblastoma and lung CSCs [41] and that the chemoresistance in colon CSCs was acquired through upregulation of DR4 [42]. These facts highlight TRAIL potential as a CSCs eliminating agent. However, in some situations where apoptotic signalling pathways are deregulated in CSCs, TRAIL-based combinational approaches are required. In CD133+ glioblastoma, T-cell acute leukaemia, and breast cancer cells, the TRAIL inhibitory cFLIPs proteins are upregulated [43]. Silencing of cFLIPs by siRNA and its downregulation by cisplatin restores cell sensitivity to death stimuli, suppressing CSC self-renewal and tumour metastasis [44–45].

Excitingly, the three musketeers of CSCs self-renewal; Wnt, Notch, and Hedgehog pathways can be knocked down using TRAIL-based combination leading to elimination of cancer recurrence and relapse.

**Figure 2.**
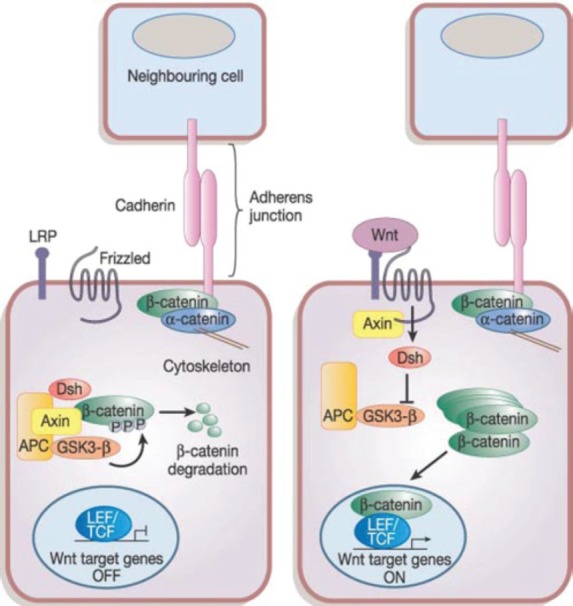
The canonical Wnt signalling pathway. Reused with permission of [121].

In the Wnt pathway, the Wnt family secreted proteins bind to specific Frizzler (FZD) receptors on the surface of CSCs activating intracellular pathways, resulting in β-catenin protein uncoupling from its degradation complex and nuclear translocation [46] as depicted in Figure 2. This nuclear β-catenin binds to T-cell factor 4 (Tcf4) resulting in target gene activation, including cyclin D1, c-Myc, and survivin allowing apoptosis escape. Treatment of triple negative breast cancer (TNBC) stem cells with cisplatin and TRAIL results in inhibition of Wnt downstream target, β-catenin, phospho β-catenin, and cyclin D1 leading to increasing apoptosis and reduced proliferation [47].

Interference of Hedgehog pathway using genetic and pharmacological tools also sensitises cells to TRAIL cytotoxicity. The Hedgehog-GLI pathway modulates DR4 expression by binding of the transcription factor GLI3 to DR4 promoter. Hence targeted knock down of GLI-3 by small interfering RNA (siRNA) resulted in restoration of DR4 expression and TRAIL sensitivity [48]. Using cyclopamine, a well-established pharmacological inhibitor of smoothened (SMO) which is the signalling component of the Hedgehog receptor complex, lead to sensitisation of cholangiocarcinoma cells to TRAIL [49]. Cyclopamine treatment downregulated cellular IAP-1 (cIAP-1) and X-linked IAP (XIAP) protein levels via the Hedgehog inhibition dependent mechanism. Also, the same study concluded that Hedgehog inhibition can promote TRAIL sensitisation independently from Bid, Bim, Bax, and Bak.

Notch is a transmembrane heterodimeric receptor present in four different forms in humans (Notch 1–4) [50]. Since all Notch receptors are activated by γ-secretase using inhibitors of this enzyme (GSIs) with TRAIL lead to sensitisation MDA-MB-231 breast cancer cells to TRAIL induced apoptosis [51]. The underlying mechanism for that synergism lies in Notch inhibition by GSIs leading to: 1) transcriptional activation of DR4 and DR5 and their overexpression via AP1 dependent mechanism, 2) downregulation of survival factors as phosphorylated form of AKT and IAP members like survivin and c-IAP-2 and 3) increase in proapoptotic elements as Noxa and members of the Bcl-2 family.

However, the complete relation between self-renewal pathways and TRAIL signalling in different types of CSCs remains a little bit ambiguous and require future analysis for better understanding.

Based on these promising effects as apoptosis inducer, scientists introduced soluble human recombinant TRAIL and agonistic monoclonal antibodies targeting either DR4 or DR5 in clinical fields [52].

## TRAIL in clinical trials

In general most TRAIL preparations have passed phase I trials with no significant toxicity and were tolerated by most of the patients. However, a literature review of phase II trials up till now shows that different TRAIL preparations did not meet the expectations and had nearly no impact when used as monotherapy or in addition to standard regimens.

A randomised phase II study was done by Soria *et al* to evaluate the efficacy and safety of dulanermin(soluble recombinant human TRAIL) combined with paclitaxel and carboplatin (PC) and bevacizumab(B) as first-line treatment for advanced or recurrent non–small-cell lung cancer [53]. The addition of dulanermin to PC and PCB had no impact on progression-free survival (PFS) and overall survival (OS).

Tigatuzumab (CS-1008) is so far the most widely assessed monoclonal antibody targeting TRAIL-R2. In 2013 a randomised phase II study was performed using tigatuzumab versus placebo as a combination therapy with carboplatin and paclitaxel in patient with advanced NSCLC, tigatuzumab did not improve the efficacy of carboplatin/paclitaxel treatment [54].

Tigastuzumab was also tested in combination with gemcitabine for treatment of metastatic pancreatic cancer, it was well tolerated during the trial and the numeric trends described in the study suggested a possible contribution of tigatuzumab to the antitumour efficacy of gemcitabine. However there were no definitive conclusions which can be drawn regarding the contribution of tigatuzumab to the observed overall response rate (ORR), PFS, or overall survival data (OSD) [55].

Mapatumumab, also called TRM1 or HGS-ETR1, is the only TRAIL-R1 antibody assessed in clinical trials. Monotherapy trials assessed its role against advanced solid tumours like refractory non Hodgkin lymphoma [56], refractory colorectal cancer [57], and stage IIIb/IV refractory non small cell lung cancer [58]. Mapatumumab was well tolerated in general, however, the results were disappointing as none of these approaches have significantly impacted the OS of patients.

Conatumumab, a humanised monoclonal agonistic antibody against TRAIL-R2, was assessed in combination with FOLFIRI chemotherapy as a second-line treatment to patients with mutant KRAS metastatic colorectal carcinoma. It showed a trend toward improvement of PFS with tolerated toxicity [59]. Another trial was done by Fuchs *et al* in which conatumunab was tested against placebo as a combination therapy with mFOLFOX6 plus bevacizumab in treatment of metastatic colorectal cancer. In contrast to a former study conatumumab did not demonstrate improved efficacy over placebo [60].

The failure of TRAIL preparations in proving a statistical benefit in OS or PFS might be attributed to pharmacokinetic properties of such preparations or inability to reach proper concentration of preparation at site of action.

Another proposed mechanism is the development of TRAIL resistance in cancer cells by one or more of the mechanisms mentioned previously.

TRAIL gene transfer appear to be a promising alternative as it results in the production of targeted stable high concentration in cancer tissues and possible bystander effects [61]. Also, the role of cancer gene therapy has been increasing specially after (T-Vec) approval by the FDA in 2015 for melanoma treatment [62]. In the following section we intend to review all the methods used for TRAIL gene delivery.

## TRAIL gene delivery system

### Viral vectors for TRAIL gene delivery

Viral vectors demonstrated more privileges over non-viral ones because of their natural efficient cell attachment abilities, entry, and highest level of transgene expression as part of the viral replication cycle [63–64].

These viral vectors are derived from adenoviruses, adeno-associated viruses, retroviruses, poxviruses, and herpesviruses with each and every one of them having its own advantages and limitations. Adenoviruses because of their relatively simple production and manipulation, high transduction efficacy without being integrated in cell genome, and ability to accommodate up to 30-kb DNA are preferred over other viral vectors. Both helper-dependent high-capacity Ad vectors that completely lack viral coding sequences or conditionally replicating Ads (also known as CRAds) driven by tumour-specific promoters in their E1A regions have been used to deliver TRAIL gene [24, 61, 65]. CRAds are dependent on coxsackie-adenovirus receptor (CAR) for cell entry. However, they can also be engineered to target alternative cell receptors that are overexpressed in tumour tissues than in normal tissues like CD46 [66]. This fact makes adenoviruses a promising vector to overcome obstacles of viral gene transduction therapy in terms of cell entry and toxicity to normal tissues. The ability of Ad vector to transfer full length of TRAIL cDNA selectively to tumour cells resulted not only in its rapid production and cancer apoptosis, but it also had bystander effect which is believed to augment its therapeutic and clinical value [67]. In the same experiment Seol *et al* [67], assumed that Ad TRAIL bystander effect is media transferable not contact mediated as demonstrated by Kagawa *et al* [61]. However, both effects are very useful in clinical situations as they can overcome any viral limited transduction efficiency in tumours. It is also to be noted that intracellular pathway of apoptosis induced by Ad-TRAIL differs from its media transferable-bystander effect where it is released passively from dying cells to interact with neighbouring cells receptors.

Another advantage of these adenoviral vectors is that they can be double armed with any gene beside the one coding TRAIL to potentiate the tumoural cytotoxicity of the cancer targeted gene virotherapy (CTGVT) or to overcome TRAIL resistance. Reconstruction and recombination of two different adenoviral vectors one carrying shRNA and the other coding TRAIL in E.coli cells resulted in single double armed vector expressing shRNA beside TRAIL [68–69]. These short hairpin RNA that can interfere with cancer cells RNA can be used to silence the expression of anti-apoptotic proteins like the IAP family member survivin [68], the nuclear protein Daxx [69], or the β-galactoside binding protein galectin-1 [70] and thereby augment the cytotoxic effect of TRAIL in cancer cells. CRAd coding TRAIL can also be loaded and double armed with a separate cassette coding Arresten gene. This dual expression can also potentiate the TRAIL induced cancer cell apoptosis as Arresten affects different cell signalling pathways to inhibit angiogenesis and tumour growth [66]. Also, double armed vectors loaded with suppressor of cytokine signalling 3 (SOCS3) has shown to reverse the resistance of HCC to TRAIL-induced apoptosis through the interaction between SOCS3 and JAK/STAT pathway. [71]

Another virus that has not been extensively studied as adenovirus for TRAIL delivery is the Newcastle disease virus (NDV). Recombinant NDV have shown oncolytic effects either alone or in combination to other genes or drugs in many pre-clinical studies [72–73] and is being considered for clinical trials now. The rNDV expressing TRAIL gene and IL-2 was found to induce apoptosis in mouse models bearing hepatocellular carcinoma and malignant melanoma successfully [74]. In late 2015, a study showed that not only TRAIL can be transmitted by rNDV but also can be used synergistically to augment the oncolytic effect of this virus as caspase-8 activated byTRAIL binding to DR4/DR5 amplifies the intrinsic mitochondrial cycle initiated by rNDV [73].

### Non viral vectors for TRAIL gene delivery

Non-viral systems for gene transfer include deliberately introducing nucleic acids into cells DNA using nanotechnology, mesenchymal cells, microparticles. The chemical methods include a variety of liposomes, peptide delivery systems, cationic polymers causing disruption of the cell membrane. Physical methods for such include electroporation or ultrasonography.

Liposomes with bioactive TRAIL tethered to their surfaces did not only act as an efficient mode of its delivery, but rather improved its cytotoxicity by clustering of DR5 on cell surface [75]. Also, cationic liposomes can be modified and engineered to act as co-delivery system double like adenoviruses to express human TRAIL gene beside other drugs to improve tumour cells apoptosis outcome. This concept was tested using liposomes double delivering TRAIL and paclitaxel in glioma and found to be an effective method of either *in vivo* or *in vitro* dual delivery [76].

The role of cationic polymers is not only restricted to TRAIL gene intra-tumoural delivery but can also be used to enhance the transfection of TRAIL encoding adenoviruses in cells that down express (CAR) by overcoming the repulsion between the epithelial cells coated with anionic glycosaminoglycan and negatively charged adenoviral surfaces [77]. The ethylene glycol diglycidyl ether (EGDE)-3,3′-diamino-N-methyl dipropylamine (3,3′) also coined as (EGDE-3,3’ ) polymer is a promising tool in this regard as it lacks the toxic effects of the most commonly used polymer polyethylenimine (pEI) and enhances the transduction of Ad TRAIL in bladder cancer cells lacking (CAR) [78].

The peptide delivery system implies the delivery of targeted genes or protein in living cells using a protein transduction domain (PTD) or cell penetrating peptides [79]. The number of available PTD is enormous and keeps growing since the region between amino acids 47–57 of the transactivator of transcription (TAT) protein of HIV-1 (human immunodeficiency virus type 1) was found to be responsible for cellular membrane penetration [80]. Arginine-rich intracellular delivery (AID) peptides were found to be successful in delivering plasmids coding human TRAIL into human lung carcinoma A549 cells [81]. Also, peptides can be used for targeted gene delivery by binding to specific integrins only expressed in cancer cells or tumour blood vessels. In this regard, ACDCRGDCFC a targeting peptide to human α_V_β_3_ and α_V_β_5_ on tumour blood vessels was fused to TRAIL forming what is called RGD-L-TRAIL and improved the therapeutic index of TRAIL *in vitro* and *in vivo* [82].

Chimeric polypeptides formed by cross-linkage of octa-D-arginine (cell penetration-facilitating group and DNA-binding moiety) and tetra-L-histidine (for the acquired endosomal buffering capacity of the polypeptide) are reported to be highly efficient vectors for TRAIL delivery.

TRAIL delivered by reducible chimeric polypeptides considerably inhibited tumour growth formation in mice xenografted with HeLa cells [83].

**Figure 3.**
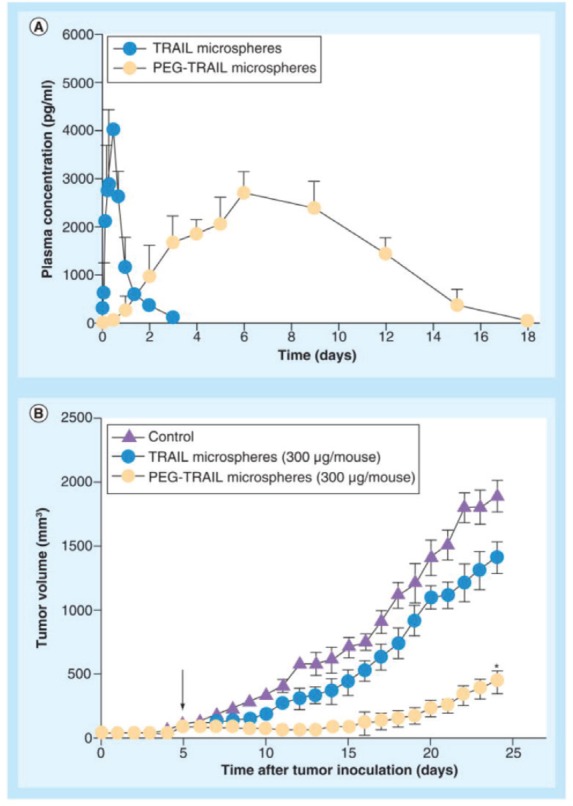
(A) In vivo pharmacokinetic profiles of TRAIL or PEG-TRAIL microspheres after subcutaneous administration (100 μg/rat; n = 5); (B) Tumour growth suppressions by TRAIL or PEG-TRAIL microspheres (300 μg/mouse, subcutaneous). Reused use permission of [122].

Microparticles formed of biodegradable polymers are also another effective mode for TRAIL delivery. PEGylated TRAIL microparticles (where Mono-methoxy PEG (mPEG) molecule is added to the microparticle) showed supremacy to regular TRAIL microparticles in terms of encapsulation efficacy and low initial burst release. *In vitro* experiments showed 15.8 % calculated burst release from PEG-TRAIL microspheres in comparison to 42.7% for TRAIL microspheres. Also, *in vivo* experiment showed 25.9% and 78.3% tumour regression for TRAIL microparticles and PEG-TRAIL respectively at 300 μg of TRAIL/mouse [84] as depicted in Figure 3.

Synthetic polyurethanes (PUs) which have the ability to incorporate urethane (carbamate) bonds into polymeric backbone were used as delivery systems as well. Paclitaxel loaded water borne polyurethane nanomicelles conjugated with TRAIL (PTX-PU-TRAIL) have been tested for efficacy against 4T1 bearing balb/c mouse model [85].

Transdifferentiation (TD)-derived induced neural stem cells (iNSCs) have been engineered with an image able fusion between TRAIL (iNSC-diTR) and Gaussia luciferase [86]. Results revealed that treating LN18 and U87 glioblastoma cell lines with conditioned media from iNSC-sTR treatment considerably enhanced activity of caspase-3/7 in a time dependent manner. Furthermore, in the treatment of mC-FL expressing U87 glioblastoma cells intra-cranially implanted in mice, the survival rate of mice treated with iNSC-sTR induced tumour regression and survival increase.

### Mesenchymal cells for TRAIL gene transfer

Mesenchymal cells (MSCs) can be isolated from either adult or fetal tissues. Their ability to migrate to tumour sites as well as given their pre-metastatic niche, low immunogenicity, easy reproduction, genetic manipulation, and modification make them a very promising tool for transgene expression [87]. These facts caused them to be extensively studied in comparison to other non-viral gene delivery systems for TRAIL delivery. However, it is to be noted that each tumour type treatment requires a specific MSCs type to achieve the desired results. This fact was concluded after Akimoto *et al* discovered that co-culturing adipose tissue derived MSCs (AT-MSCs) with GBM cells induced tumour vascularisation and proliferation, while co-culturing with human umbilical cord blood-derived MSCs (HUMCs) inhibited tumour growth [88]. Also, the mode of delivery of MSCs harbouring TRAIL (MSCs-TRAIL) affected their tumour incorporation as it is seen that IV delivered MSCs incorporated more in tumours than intrapleurally-delivered MSCs [89].

Virally transfected or non-viral gene modifiedMSCs encoding soluble TRAIL were tested against different types of tumours. It was concluded that AT-MSCs-TRAIL were effective against sarcomas by inducing apoptosis in different osteosarcoma, Ewing’s Sarcoma (ES), and rhabdomyosarcoma cell lines *in vitro* and in pre-established ES xenotransplants *in vivo* [90]. Human malignant mesothelioma responded as well to the antitumour effects of TRAIL-expressing MSCs derived from human bone marrow, and the inflammatory tumour environment *in vivo* was successfully reduced after their administration [91]. An antitumour activity was also noted in TRAIL sensitive intracranially implanted medulloblastoma and in TRAIL-resistant MBs pretreated with MS-275 after human MSCs-TRAIL treatment [92]. Also, non-viral TRAIL-engineered AD-MSCs were capable to migrate towards multiple myeloma cells and induce tumour cytotoxic effects [93]. Also, MSCs transducing TRAIL were able to target a subgroup of cancer stem cells resistant to common oncological treatments known as side population (SP) [94].

Engineered human umbilical cord mesenchymal stem cells (HUMSCs) to secrete soluble TRAIL via adenoviral transduction mediated by cell-permeable peptides were found to be very effective in reduction of glioma in *in-vitro* cultures and also to migrate efficiently towards human glioma cells and prolong the survival of athymic nude mouse bearing intracranial xenografts of human glioma [95]. Also, Ad.sTRAIL(DR4) transfected MSCs exhibited tumour inhibition against pancreatic cancer cells PancTu1, while colorectal cancer cells HCT116 responded to MSCs transfected with Ad.sTRAIL(DR5) [96]. Adenovirus was not the only virus that was used to transfect MSCs. The human MSCs transduced with a lentiviral vector encoding TRAIL successfully migrated to malignant mesothelioma [5] and to hepatocarcinoma driven by AFP promoter [97] and induced tumour cells apoptosis. Also, rat bone marrow MSCs encoding s TRAIL via lentiviral vector transduction were able to induce hepatocarcinoma growth inhibition in mouse models [98]. Using lentivirus-TRAIL, transduced MSCs induced significant apoptosis in different types of cancer cell lines as well as showed remarkable reduction in metastatic tumour burden with frequent eradication of metastasis in murine lung metastasis mode [99]. Interestingly, herpes simplex virus (HSV) type I amplicon vector secreting the trimeric form of TRAIL was used to engineer MSCs and showed increased survival of mouse models with intracranial glioma [100]. It is to be noted that combinational approaches with different chemotherapeutic agents as cisplatin [101], lipoxygenase inhibitor MK886 [102], and temozolomide [103] enhanced the apoptotic effect of MSCs-TRAIL against different cancer types.

Recently a new role for MSCs has been explored. Kaczorowski *et al*, used MSCs isolated from bone marrow as delivery vectors for oncolytic adenovirus (OAd5/3-TRAIL). They proved that this method of viral delivery was more efficient and tumour selective than the virus alone and had a tendency towards cancer stem cells targeting [104].

Knowing the selective MSCs homing ability to specific types of cancer cells can subsequently improve TRAIL delivery and apoptosis induction. Intravenously injected HUMSCs have been shown to migrate to lung cancer after 24 hours via MCP-1/CCR2 transduction cascade [30]. This cascade consists of overexpression of MCP-1 on lung cancer A549 cells and expression of its receptor CCR2 on the surface of HUMSCs. These TRAIL-expressing HUMSCs dramatically enhanced apoptotic rate in tumour cells and prolonged survival of xenografted mice.

### Nanotechnology

**Figure 4.**
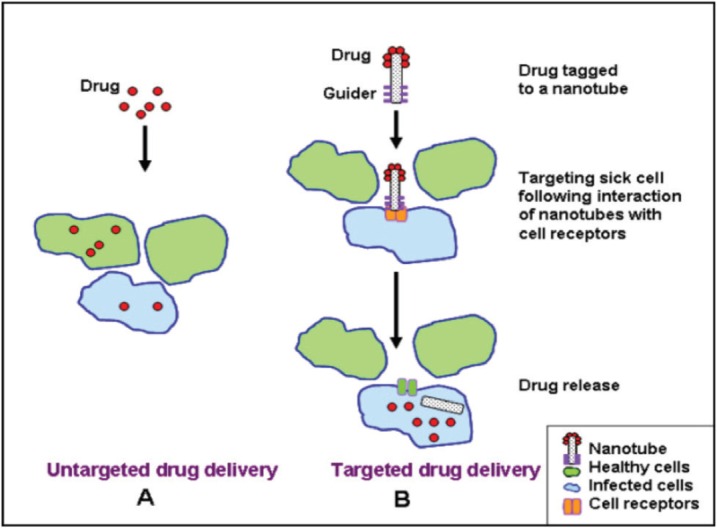
Comparison between untargeted and targeted drug delivery using a NP.

Recently,different modalities of colloidal and non-colloidal nanoparticles (NPs) with different biological properties and compositions have been investigated for drug/gene delivery in cancer therapy as depicted in Table 1. Nanoparticles are taken up by cells more efficiently than larger micromolecules and therefore could be used as effective transport and delivery systems. Nanovectors in comparison with free antitumour drugs showed better therapeutic results and enhanced intra-tumoural accumulation [105] as depicted in Figure 4. Their effectiveness is attributed to their small size allowing penetration of cell membranes, proteins stabilisation, and binding and escaping lysosomal degradation after endocytosis.

Also, these nanosystems showed:

1. Passive accumulation into tumour target sites through a phenomena known as enhanced permeability and retention effect (EPR) because of lack of functional lymphatics and tumour angiogenesis [106].
2. Ability of targeted delivery by binding to specific cell receptors when modified with a guider ligand thus sparing the normal tissues and enhancing the drug/gene therapeutic index [106].
3. Modification of drug pharmacokinetics and biological distribution [107].
4. Prolonged presence in the bloodstream when their surface is coated with polyethylene glycol (PEG) molecule which helps escaping phagocytosis by the reticuloendothelial system cells [108].

All these characteristics made nanovectors a very promising vector for targeted gene therapy and efficient TRAIL delivery was achieved using different nanotechnology-based systems.

A NP with an iron oxide core surrounded by a layer of chitosan–polyethylene glycol (PEG)-grafted polyethyleneimine (PEI) copolymer (CP-PEI) and modified with chlorotoxin (CTX) for glioblastoma targeting,successfully delivered plasmid coding human TRAIL to human T98G GBM cells. Moreover to *in vitro* increased apoptosis, complete regression of T98G-derived flank xenografts in nude athemic mice was reported after NP-TRAIL-CTX treatment [109].

Also, magnetic ferric oxide nanoparticles (NPs) conjugated withTRAIL demonstrated apoptotic activity against different human glioma cells and germline stem cells (GSCs). A targeting ability to U251 cell-derived glioma xenografts associated with tumour volume decrease and increased survival of animal models was also reported with these NPs [110].

A dendrimer, which is a nanosized polymer formed from a central core surrounded by successively added layers of branching structures called dendrons, has been used for transgene expression. An engineered triazine—which binds DNA through hydrogen bonds—modified on a polyamidoamine (PAMAM) dendrimer was used successfully to deliver TRAIL coding plasmid to osteosarcoma cell line MG-63. It also showed beside apoptosis induction low cytotoxicity and high transfection efficacy in comparison to other polymers [111]. These dendrimers do not only serve as transmission vectors but also can be used for targeted gene delivery. Transferrin (Tf)-modified (PAMAM) dendrimer which has great brain targeting efficiency [112] was used to deliver (hTRAIL)-encoding plasmid in C6 cells and showed enhanced cytotoxicity, glioma targeting, and enhanced permeability and retention (EPR) effect in comparison to non modified NPs [113].

Interestingly, the NPs can be also designed to act as a dual delivery system making them an optimum vector paving the path to optimum TRAIL gene-based therapies. The advantage of any chemotheraputic combination with TRAIL does not only aim to enhance its cytototoxic effects, but rather to achieve better safety profiles by dose reduction resulting from combination synergism.

A polylactic-co-glycolic acid (PLGA)-based multi-layered nanoparticles (MLNP) was used as co-delivery system for camptothecin (CPT) a topoisomerase-1 inhibitor and plasmid coding rhTRAIL. Beside the enhancend *in vitro* and *in vivo* apoptosis in different cell lines treated by these MLNPs, this co-delivery system succseded in reducing the CPT and pTRAIL dosing by 3.1–15 fold and 4.7–8.0 fold respectively because of their synergestic action [114].

Another design of NPs that can serve as one pot, delivering combinational formulation of genes and chemotheraputic agents, is the albumin-bound nanoparticle (HSA-NP). Pancreatic cancer Mia Paca-2 cells showed apoptosis following treatment with HSA-NP delivering paclitaxel and TRAIL [115] while HCT116 coloncancer cell were inhibited by HSA-NP carrying doxorubicine and TRAIL [116].

In accordance with the approach of drug and gene co-delivery via cationic liposomes, nanoscale designed liposomes were used to deliver doxorubicin and TRAIL to produce synergistic apoptotic effct in non-small cell lung cancer (NSLC) [117] and glioblastoma multiforme (GBM) cancer cells [118]. Intrestingly,double-genetherapy for coloncancer was mediated by a nanoliposome containing tyrosinekinase receptor 3 ligand and TRAIL-encoding plasmids [119]. This double design offers a new route to combined gene therapy and immunotherapy for cancer treatment.

There is a recent report highlighting significance of transformable core-shell-based nanocarrier (CS-NG) as an effective system to deliver TRAIL and anti-angiogenic agents [120]. CS-NG enzymatically assembled into micro-sized extracellular depots at the site of tumour with the help of an enzyme hyaluronidase, frequently overexpressed in the tumour microenvironment. Structurally, CS-NG is composed of core-NG (C-NG) loaded with therapeutics, shell-NG (S-NG) with encapsulated transglutaminase (TG), and human serum albumin (HSA), coated on the surface of C-NG. Acid-degradable polymers for delivery of therapeutically effective agents have attracted considerable attraction.

CS-NG is equipped with an acid-degradable modality, and therefore it effectively releases combinatorially delivered cilengitide (antiangiogenic agent) and TRAIL to endothelial cells at the acidic tumour microenvironment and membrane of cancer cells respectively. This method showed effectiveness against breast cancer MDA-MB-231 cells.

## Conclusion

Induction of apoptosis could be the ultimate approach for different types of cancer treatment. TRAIL is a powerful molecular agent for apoptosis induction. The recent approval of gene therapy in the form of (T-Vec) for melanoma treatment paves the pathway towards succesful future gene therapy trials. Incorporating TRAILin future oncolytic viruses, based on its selective potency and interaction with CSCs, will definitely enhance their outcome. Using hybrid gene delivery systems, a safe viral vector targeting cellular integrins and receptors of CSCs through peptide targeting ability can be achievied. Other options for promising gene therapy clinical trials approaches will include TRAIL transgene delivery by mesenchymal stem cells and nanoparticles. Likewise, combining TRAIL with chimeric antigen receptor (CAR) T-cell, could augment the effect of this approach. To this end, TRAIL usage could tackle a myriad of oncological conditions thereby achieving a shift in the paradigm of cancer treatment.

## Figures and Tables

**Table 1.** Different types of nanoparticles investigated for drug/gene delivery.

Particle class	Materials	Application
Natural materials or derivatives	Liposomes Chitosan Gelatine Dextrane Starch Alginates	Drug/gene delivery
Dendrimers	Branched polymers	Drug delivery/gene delivery
Polymer carriers	Block copolymers Polylactic acid Polycaprolactone Polyethyleinemine Poly(cyano)acrylates	Drug/gene delivery
Various	Silica-nanoparticles Mixtures of above	Gene delivery
